# Influence of Toothbrush Abrasion and Surface Treatments on Roughness and Gloss of Polymer-Infiltrated Ceramics

**DOI:** 10.3390/polym13213694

**Published:** 2021-10-27

**Authors:** Nawaf Labban, Mohammad D. Al Amri, Sarah M. Alnafaiy, Saleh M. Alhijji, Mohammad A. Alenizy, Mounir Iskandar, Sabrina Feitosa

**Affiliations:** 1 Department of Prosthetic Dental Sciences, College of Dentistry, King Saud University, Riyadh 11545, Saudi Arabia; malamri@ksu.edu.sa (M.D.A.A.); saranafaiy@yahoo.com (S.M.A.); 2 College of Applied Medical Sciences, King Saud University, Riyadh 11545, Saudi Arabia; smalhijji@ksu.edu.sa; 3 School of Dentistry, Indiana University, Indianapolis, IN 46202, USA; 4 Department of Restorative Dental Sciences, University of Hail, Hail 55475, Saudi Arabia; Dr.m.alanazi@gmail.com; 5 Private Practice at Radiance Dentistry, Irving, TX 75063, USA; mounirshawki@yahoo.com; 6 Department of Biomedical Sciences and Comprehensive Care, Division of Biomedical and Applied Sciences, Indiana University School of Dentistry, Indianapolis, IN 46202, USA; sfeitosa@iu.edu

**Keywords:** surface gloss, surface roughness, CAD/CAM, polymer-infiltrated ceramic, citric acid, abrasion

## Abstract

The aim of this study was to compare the surface roughness and gloss of polymer-infiltrated ceramics after simulated in vitro toothbrushing in different storage mediums. Four polymer- infiltrated ceramics were evaluated, Lava ultimate (LU), Vita enamic (EN), Shofu (SH), and Crystal ultra (CU). The control group was a feldspathic ceramic, Vita Mark II (VM). One hundred and twenty specimens (12 × 14 × 2.5 mm) were prepared using a precision saw. For each material (n = 24), the specimens were allocated into two groups, polished and stained. The specimens of each group were stored (for 7 days) in either citric acid (0.2N) or distilled water. Data for surface gloss (ΔE*_SCE-SCI_) and roughness (Ra) were evaluated before (baseline) and after simulated toothbrushing. For toothbrushing simulation, a toothpaste slurry containing a toothpaste of 100 relative dentin abrasion (RDA) and 0.3 ml distilled water was used for 3650 cycles (7300 strokes) for each specimen. Data were analyzed using t-test and ANOVA. A *p*-value of ≤ to 0.05 was considered significant. The highest mean value of surface gloss was identified in CU (stained—water) (4.3 (0.47)) (ΔE*) and EN (stained—acid) (4.3 (1.00)) (ΔE*) specimens, whereas the lowest mean value was shown by SH (stained—acid) (2.04 (0.42)) (ΔE*) samples. The highest mean value of surface roughness was observed in SH (0.40 (0.99)) Ra (stained—acid) whereas the lowest in VM (0.13 (0.039)) Ra (polished— water). A significant difference (*p* < 0.05) was observed in surface roughness and gloss between the materials with simulated toothbrushing, except in VM and LU, respectively. Therefore, it can be concluded that simulated toothbrushing impacts on surface roughness and gloss, irrespective of the storage medium.

## 1. Introduction

Aesthetic maintenance of dental restorations is a challenge faced in dental practice. Continuous efforts have been made to improve restorative surface qualities such as gloss and surface roughness. In a study by Gracis et al., resin matrix ceramics were defined as materials with an organic matrix highly filled with ceramic particles [1]. They classified highly cured resin matrix reinforced with approximately 80% nano-ceramic particles as resin nanoceramics. Another type of polymer-infiltrated ceramic is “Glass ceramic in resin interpenetrating matrix”, which is comprised of a dual network of feldspathic ceramic and a polymer network [1]. These different types of contemporary ceramics–polymer materials are regarded as polymer-infiltrated ceramics, as they are comprised of a combination of polymers and ceramics. For superior surface outlook, 15% of clinicians have adopted polymer-infiltrated ceramic materials that combine ceramic and resin composites [2,3]. Unlike resin composites, resin-infiltrated ceramics are composed of two interlocking structures: low-viscosity polymer infiltrated into a sintered glass–ceramic network [3]. This fusion of polymers with ceramics contributes to substantial mechanical strength with a lustrous, glossy, and smooth surface [3]. Thus, wear resistance, chemical stability, and aesthetic appeal promote the adoption of polymer-infiltrated ceramics in clinical practice.

The quality of the surface is one of the critical factors in determining clinical success and longevity [4,5]. Surface roughness and gloss are two crucial factors responsible for the aesthetic appearance of a restoration [6]. The primary factors in controlling esthetic restorative outcomes are light transmission and the translucency of the material [7]. It has been suggested that rough surfaces reflect light in several directions because the surface behaves as many tiny surfaces as possible, thereby compromising the esthetic outcomes [7]. Surface roughness is commonly measured as the roughness average of a surface’s measured microscopic peaks and valleys denoted as Ra. Studies have shown that the long-term interaction of the oral environment with a combination of mechanical factors such as toothbrushing and dental abrasives contributes to the surface roughness and loss of gloss with time [8,9]. Therefore, irrespective of the surface treatment employed to improve the surface quality of the restoration, the oral environment plays a critical role in surface degradation. The change identified is due to the weakening of the resin bond, the wearing of fillers, and the degradation of the matrix [4,10].

Nevertheless, the physiochemical fusion of the ceramic and resin matrix with varying ratios has evolved this vulnerability. The optical phenomenon of the gloss effect is achieved through a reflection of light that depends on the surface capacity and surface roughness governed by the composition [11,12]. The glassy network in ceramics has demonstrated greater power to allow reflected light to pass; hence, the aesthetic stability of the composites is lower than that of the ceramics [2,5].

Nevertheless, to enhance the surface quality and appearance, dentists follow three steps in their clinical practice: polishing, staining, and glazing [12]. To reduce the surface energy, enhance the luster, and mimic natural tooth appearance, the dentist recommends restorative polishing with varying levels of abrasives and composition. Studies have shown that polymer-infiltrated ceramics can retain surface gloss even under vigorous brushing; however, surface roughness is inevitable [11,13]. Therefore, authors have reported an increase in bacterial retention in the form of plaque due to increased surface roughness, which is expected to also contribute to loss of gloss and staining of the restoration [14].

Studies have shown that dentists prefer monolithic CAD/CAM materials to resin-based restorations due to their resemblance to natural teeth and slower surface degradation processes [15,16]. In addition, it has been reported that the size and shape of the composite fillers contribute to the surface morphology, which influences material characteristics [9,17]. In the presence of low-viscosity polymer infiltrated into sintered glass–ceramic, and further incorporation of nanoparticles, the polymer-infiltrated ceramics may result in a smoother and glossy surface finish, with reduced chances of surface deterioration over time. To our knowledge, limited data are available regarding polymer-infiltrated ceramics correlation with surface roughness and gloss under the influence of toothbrushing in the presence of oral fluids. The null hypothesis was that the surface roughness and gloss would not be significantly influenced by toothbrush abrasion of polymer-infiltrated ceramics in the presence of different storage mediums. Thus, the study aimed to compare the polymer-infiltrated ceramic surface roughness and gloss after simulated in vitro toothbrushing in other storage mediums.

## 2. Materials and Methods

Four resin polymer-infiltrated ceramic materials including Lava Ultimate-LU (3M ESPE), Vita Enamic- EN (Vita Zahnfabrik), Shofu HC- SH (Shofu Inc., Kyoto, Japan), Crystal Ultra—CU (Digital dental), and one feldspathic ceramic (Vita mark II- VM (Vita Zahnfabrikas)) as a control, were compared. Table 1 presents the composition and manufacture details of the materials. A total of 120 specimens were cut into blocks using an automated Isomet 1000 precision saw (Buehler, bluff, Chicago, IL, USA) at 500 RPM with a 102 mm × 0.3 mm (diameter x thickness) from CAD/CAM block of each material (n = 24) with a precise measurement of 12 × 14 × 2.5 mm. Each specimen block was smoothed with a grinding machine (Tegra Pol 15/Tegra Pol 1, disc speed 300 RPM for 1 min with 25N and water coolant, Buehler, Chicago, IL, USA) under a water coolant to a standardized thickness of 2.5 mm. Twenty-four specimens in each material were divided into polished (n = 12) and stained (n = 12).

Half of the total specimens (n = 60), corresponding to five different materials, were polished using a disk system. Each specimen surface was polished with silicon carbide paper (LECO spectrum system, Shofu, Koyoto, Japan) with extra grit in ascending polishing grade (400, 600, 800, 1000 and 1200) under continuous water spray. This was followed by applying diamond polishing paste (9.6, 3.1 um) (Meta Di Supreme, Buehler Co, Chicago, IL, USA). The other half of the specimens (n = 60) was left untreated. Staining for each material was performed according to the manufacturers’ instructions, presented in Table 2.

Furthermore, each group was divided into two subgroups, depending upon the storage medium used (0.3% citric acid pH 3.2 and distilled water) [18]; pH was measured using a pH-measuring probe (Accumet AB15 Basic pH meter; Fisher Scientific, Waltham, MA, USA). Each specimen block was immersed for seven days in the designated storage medium at room temperature, followed by 10 min of thorough wash (only in the citric acid subgroup).

### 2.1. Surface Gloss

Surface gloss (n = 120) was measured using a reflection spectrophotometer (CM-2600 d, Konica Minolta Sensing, Inc., Osaka, Japan) over a black and white background with Specular Component Excluded (SCE) and Specular Component Included (SCI) geometries (ΔE*_SCE-SCI_). The spectrophotometer has a measuring window of 8 mm in diameter, which focused on the specimen’s center. For each sample, two measurements were recorded: before, and after the simulated brushing. Daylight was excluded during the spectrophotometer analysis. A single operator performed all measurements.

### 2.2. Surface Roughness

Surface roughness (n = 120) was measured before and after the simulated brushing using a 2D contact profilometer (Surtronic S-100 series, Taylor Hobson, Leicester, UK), with the pointed probe placed in the center of the specimen to detect the surface roughness (Ra) in microns. Five measurements were performed for each specimen at five specific locations. The profilometer was calibrated before each group’s assessment according to the manufacturer’s recommendations. The diamond stylus at 90° stylus angle and 2 μm radius was run at a constant speed across each specimen with a 0.7 N force.

### 2.3. Simulated Toothbrushing

After recording the baseline measurement of each specimen for gloss and surface roughness, simulated toothbrushing was performed using a custom-made V-8 toothbrushing machine (ZMB 8, University of Zurich) using a toothbrush (Oral-B, P40, Procter and Gamble, Cincinnati, OH, USA), at a standardized pressure of 2.5 N. Both the specimen and the toothbrush’s head were submerged and covered with a toothpaste slurry mixture containing toothpaste of 100 RDA (relative dentin abrasion) and 0.3 mL distilled water. Then, each specimen underwent artificial toothbrushing for 3650 cycles (7300 toothbrushing strokes). After each surface was brushed with 7300 strokes, the block was rinsed with distilled water for 10 min to remove any residual smear layer. The toothpaste slurry was replaced every three hours. Study samples were assessed using scanning electron microscopy (SEM) for qualitative surface analysis before and after toothbrushing. Samples were placed on the aluminum stubs and sputter-coated with a gold layer with a sputter-coater (Baltec sputter, Scotia, NY, USA). SEM micrographs were recorded at an accelerating voltage of 10 kV in a SEM (FEI Quanta 250, Scanning Electron Microscope, Wicker, OR, USA) at different magnifications.

### 2.4. Statistical Analysis

Statistical analysis of collected data was performed using software (SPSS 21.0, IBM, Armonk, NY, USA). The normality of the data was assessed using a Shapiro–Wilk test. A paired independent t-test was employed to compare before and after the effects of simulated toothbrushing (*p* = 0.05). Moreover, *t*-test, ANOVA and post hoc multiple comparisons tests were used to analyze the difference between the subgroup in each material and among the materials. A *p*-value of ≤ to 0.05 was considered significant.

## 3. Results

Normal distribution of the data was obtained. The data comparisons were performed to explore the influence of material type, surface treatment (stain and polish), and storage medium (water and acid) on the gloss and roughness of specimens.

### 3.1. Surface Gloss (ΔE*_SCE-SCI_)

The highest mean value of surface gloss (ΔE*_SCE-SCI_) was identified for groups CU (stained—water) (4.3 (0.47)) ΔE and EN (stained—acid) (4.3 (1.00)) ΔE, whereas the lowest mean value was shown by group SH (stained—acid) (2.04 (0.42)) ΔE. Toothbrushing exhibited a significant reduction of gloss among all materials (*p* < 0.05) except LU (Table 3). In the EN group, specimens treated with stain and stored in acid showed significant gloss reduction due to brushing. Loss of gloss due to toothbrushing among the materials was irrespective of the storage medium and surface treatment (stain-polish) regimes. However, among the stained material specimens stored in water, only SH group showed loss of gloss due to brushing (Table 3).

**Figure 1:**
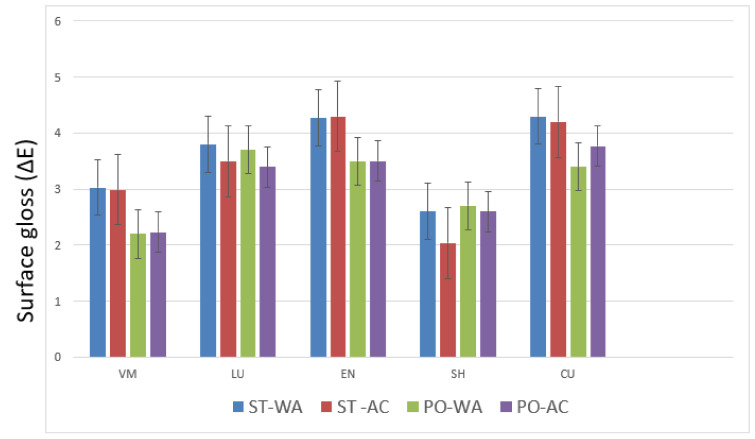
Surface Gloss (ΔE*_SCE-SCI_) comparison among the study materials based on surface treatment and storage medium employed (bars represent mean and standard error of gloss values). Intervals on the graph represent the range of surface gloss from the dataset observed.

Among different material groups, mean surface gloss loss was significantly different (*p* < 0.05) (Figure 1). The minimum and maximum surface gloss loss due to toothbrush abrasion was observed in VM (2.2 (0.51)) ΔE and EN (4.3 (1.00)) ΔE specimens, respectively (Table 3 and Figure 1). The surface gloss loss among LU (3.65 (0.55)) ΔE, EN (3.91 (0.59)) ΔE, and CU (3.92 (0.43)) ΔE specimens were statistically comparable (*p* > 0.05) (Figure 1). Likewise, overall surface gloss loss between groups VM (2.61 (0.49)) ΔE and SH (2.49 (0.34)) ΔE were also comparable (*p* > 0.05) (Table 4). However, overall surface gloss for groups LU (3.65 (0.55)), EN (3.91 (0.59)) ΔE, and CU (3.92 (0.43)) ΔE was significantly higher (*p* < 0.05) than groups VM (2.61 (0.49)) ΔE and SH (2.49 (0.34)) ΔE, respectively (Table 4). Material had a statistically significant influence on surface gloss (*p* = 0.001).

Regarding surface treatment, stained specimens after brushing showed significantly higher loss of surface gloss than polished specimens among the VM, EN, and CU groups (*p* < 0.05). However, among specimens in the LU and SH groups, surface treatment did show a comparable influence (*p* > 0.05) (Table 4). Overall, surface treatments did not show a statistically significant effect on surface gloss (*p* = 0.77). Although a majority of material groups showed higher surface gloss for materials stored in water in comparison to citric acid in respective surface treatment sub-groups, there was no significant influence (*p* = 0.16) of storage medium (water or citric acid) on the surface gloss of different materials (Table 4).

### 3.2. Surface Roughness (Ra)

The means and standard deviations of the surface roughness for the study materials under different surface treatment and storage mediums are presented in Table 5. The highest mean value was observed in the SH group (0.40 (0.99)) Ra (stained—acid), whereas the lowest in VM (0.13 (0.039)) Ra (polished—water) group. Toothbrush abrasion significantly influenced surface roughness (*p* < 0.05) among the materials compared, except for VM (control) in the stained subgroups (0.16 (0.044)) Ra. Therefore, regardless of the surface treatment and storage medium employed, toothbrush abrasion increased surface roughness in the tested materials.

**Figure 2:**
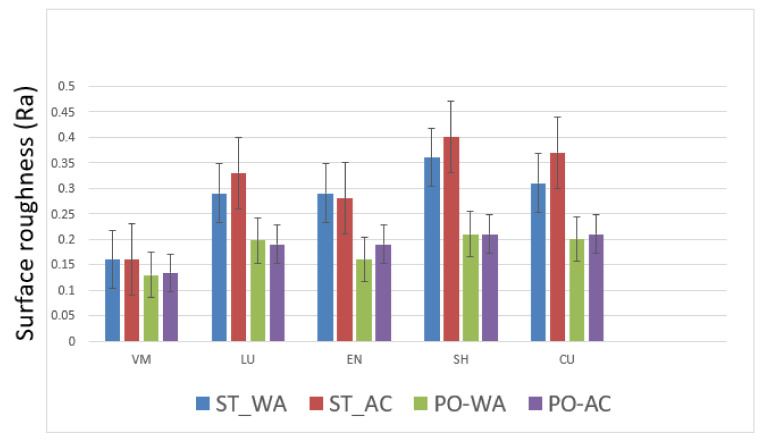
Surface roughness (Ra) comparison among the materials based on surface treatment and storage medium employed; bars represent mean and standard error of roughness values. Intervals on the graph represent the range of surface roughness from the dataset observed.

**Figure 3:**
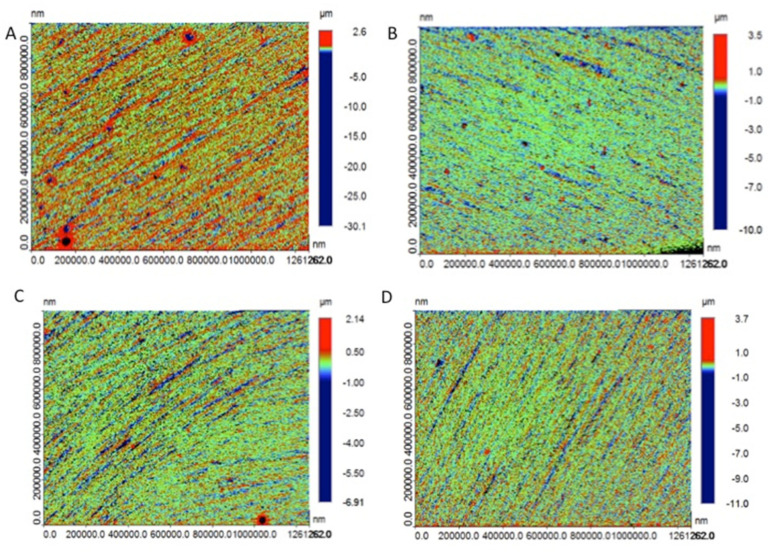
Presenting surface roughness micrographs for sample-stained SH in acid, before brushing (**A**) and after brushing (**B**). Stained samples of VM in acid, before (**C**) and after brushing (**D**).

All the polymer-infiltrated ceramics demonstrated a significant difference (*p* < 0.05) among each subgroup (Table 6 and Figure 2). The maximum mean value sought among the materials was in the stained acid subgroup for SH (0.40 (0.99)) Ra. In the SH group, the comparison between the stained acid (0.40 (0.99)) Ra and the polished acid (0.21 (0.022)) Ra group showed a significant difference in surface roughness (*p* < 0.05). However, comparable results were observed in SH (0.21 (0.022)) Ra, LU (0.19 (0.04)) Ra, VM (0.13 (0.038)) Ra, and CU (0.205 (0.01)) Ra in the polished water and acid group. The Ra micrographs before and after brushing are shown in Figure 3A–D.

In regard to material comparison, the surface roughness of four polymer-infiltrated ceramics, LU (0.25 (0.061)) Ra, EN (0.23 (0.048)) Ra, SH (0.29 (0.061)), and CU (0.27 (0.057)) Ra was significantly different for the VM (control) (0.145 (0.041)) specimens, with the lowest mean value in each subgroup (Table 6 and Figure 2). Material had an overall significant influence on surface roughness (*p* = 0.031). Furthermore, statistically, SH displayed higher (*p* < 0.05) surface roughness (0.29 (0.061)) Ra than EN specimens (0.23 (0.047)), however it was comparable to LU (0.25 (0.061)) Ra and CU (0.27 (0.057)) Ra respectively. Moreover, the surface staining presented higher surface roughness (*p* < 0.05) compared to the polished surfaces, irrespective of the storage medium (citric acid or water) used. Therefore, the surface roughness is significantly influenced by the surface treatment (stained or polished) (*p* = 0.02), compared to the storage medium (*p* = 0.071) (citric acid or water) (Table 6)

**Figure 4:**
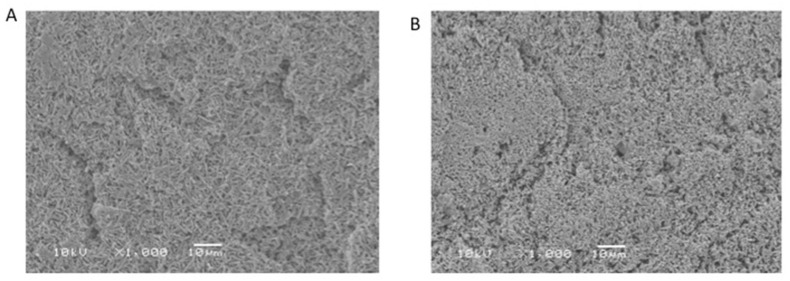

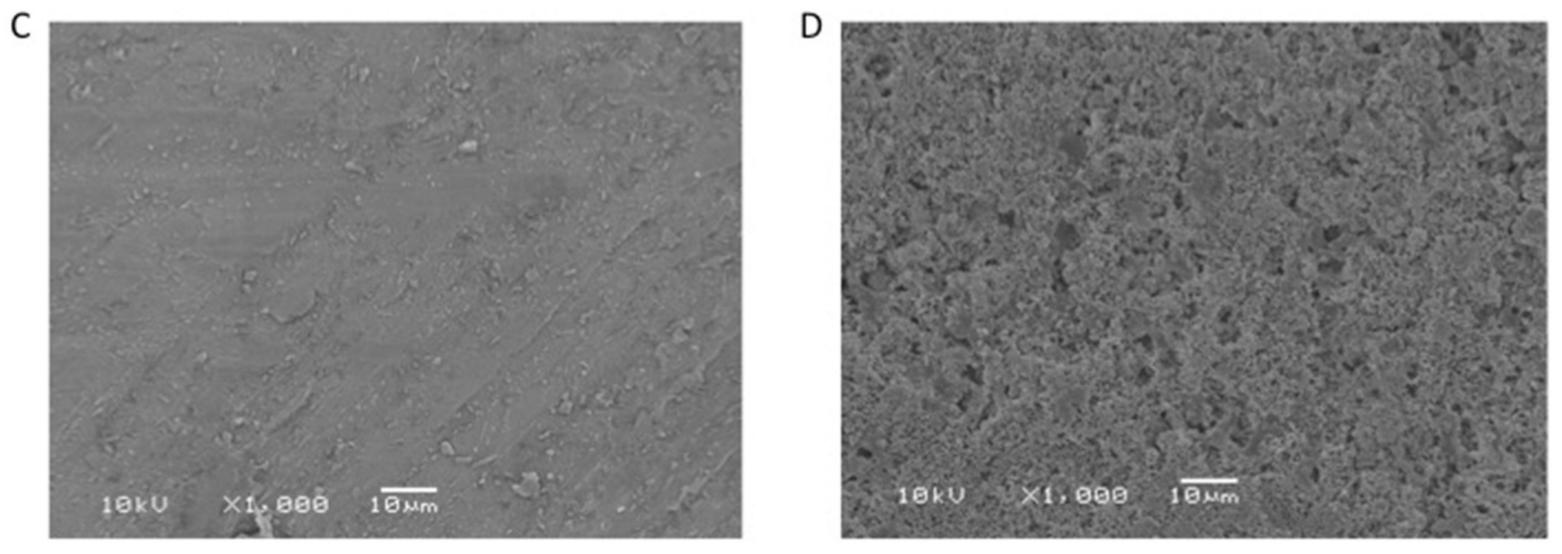
SEM micrographs presenting the quality of surfaces for material samples (stained VM in water), before brushing (**A**), after brushing (**B**); stained CU in water, before brushing (**C**) and after brushing (**D**).

Qualitative analysis of the sample surfaces is presented in Figure 4. Before brushing, samples in the control group (Figure 4A) at 1000× magnification showed presence of needle like silica crystals on the surface, with minor crevices. However, post toothbrushing, not much difference was observed, with the presence of a few more patches of removed crystal and ceramic matrix. The crevices were more significant in the samples of VM after brushing (Figure 4B). Samples for the CU material showed a smooth matrix-covered surface, with no particles observed (Figure 4C). However, after brushing abrasion, the samples showed exposed ceramic crystals embedded in the resin matrix, with areas of lost crystals and lost resin (Figure 4D).

## 4. Discussion

The present study evaluated the effect of toothbrush abrasion and acidic storage on surface gloss and roughness in polymer-infiltrated ceramics (Lava Ultimatum, Vita Enamic, Shofu, and Crystal Ultra) in comparison to a feldspathic ceramic (Vita M block II). The study demonstrated that abrasion increased surface roughness and compromised surface gloss among all polymer-infiltrated ceramic materials. Surface roughness among polymer-infiltrated ceramics was significantly higher with toothbrush abrasion as compared to conventional glass ceramic. Most of the polymer-infiltrated ceramics (except SH) showed higher loss of gloss from abrasion compared to conventional glass ceramic.

The literature states that dental restorations are subjected to various influencers that affect the restoration’s surface quality, such as thermal, chemical, and mechanical influencers [3,19]. The complex interaction of food, beverage, and saliva with toothbrushing has previously demonstrated degradation of dental restoration resulting in low durability. Clinicians’ practice of surface staining and polishing of the CAD/CAM polymer-infiltrated ceramics presented a lower risk of dental restoration deterioration [20]. The present study demonstrated that simulated toothbrushing influenced the gloss effect and surface roughness, with different intensities in all the materials, irrespective of surface treatment and storage medium. There are many explanations for the change in surface roughness and gloss under the influence of different treatments for polymer-infiltrated ceramics.

To standardize the obtained results, the present study used a reflection spectrophotometer (gloss) and 2D profilometer (roughness), peculiar for precise quantitative analysis for measuring a restricted area [21]. Surface roughness is a primary surface quality that indicates the hardness of the surface and material brittleness [6,22]. In the present study, the SH group demonstrated the highest value for surface roughness in response to toothbrush abrasion, indicating the material’s weak resistance to wear compared to the control group (VM). However, the storage medium did not show a significant negative impact compared to the brushing effect that equally affected all the materials’ surface roughness, irrespective of surface treatment. Studies have shown that surface roughness is based upon the chemical composition of materials and medium exposure [21,23]. The inherited physical property of each material reflects the type of bond strength between the organic and inorganic fillers [23]. The presence of an acidic medium weakens the resin matrix and degrades the inorganic fillers, resulting in a softening and erosion of the surface. The literature has stated that the fillers’ size and composition in polymer-infiltrated ceramic materials correlate with surface roughness [23,24]. Regardless of the storage medium and surface treatment, the reduced size of the fillers contributes to the creation of smaller voids and homogenous abrasion [7], further reducing the chances of surface roughness and loss of gloss. Among the materials used in the present study, CU has the largest filler size compared to the other materials; as CU also showed high surface roughness among the stained specimens, this can be attributed to the filler size, as suggested in previous studies [4,6,11]. However, as the reflectivity of the samples was not assessed in the present study, the above-mentioned relation of filler size and surface roughness of polymer-infiltrated ceramics needs validation from further experiments.

The inorganic content was significantly different in all the polymer-infiltrated ceramic, with Vita Enamic greater than Lava Ultimate, and CU having higher inorganic content than Shofu Block HC. Studies have shown controversial outcomes on the correlation of filler content and surface roughness; however, filler size, filler form, and polymeric matrix played an essential role in influencing the surface quality of the polymer-infiltrated ceramics [25,26]. Moreover, the loss of gloss is influenced by silanization of fillers, size, form, and resin matrix. Thus, when the filler particles are harder than the surrounding resin matrix, the simulated toothbrushing abrades the matrix easily, leading to increased surface roughness and gloss. On the other hand, authors have reported that a homogenous mixture of fillers in the material results in a consistent form of abrasion under toothbrushing; thus, the surface maintains its gloss and shows lower surface roughness [26]. In the author’s opinion, this is the possible reason for the overall significant influence of roughness and the low influence on gloss due to toothbrush abrasion, as observed in the present study [27]. This is also reflected in the low surface roughness of glass–ceramic (VM), compared to the high roughness to abrasion in polymer-infiltrated ceramics in the present study.

The finishing surface treatments of dental restorations are responsible for the final appearance of the restoration and the surface quality. The lust of gloss achieved often depends upon composition, filler size, and type of surface treatment. Studies have demonstrated that materials such as LAVA Ultimate are composed of discrete and clustered nanoparticles whose size is smaller than the wavelength [12,28]. Studies have suggested that filler size smaller than light wavelength allows the reflection of light fully, without influencing the gloss effect [25]. Nevertheless, compared to polymer-infiltrated ceramics (LU 47.1%), feldspar ceramics (control Vita Mark II, 50.8%) allow more penetration of visible light [24]. Hence, the present study accords with the assessments of previous studies. Similarly, to reduce surface roughness, emphasis has been placed on the type of surface treatment employed in the final stage: polished or stained. According to Trauth et al. [25], polishing the final product relieves the voids and flattens the final layer for better light reflection. In another study by Dal Piva et al., staining of CAD–CAM ceramics was employed for assessing comparative wear resistance [29]. It was observed that hybrid ceramic showed a higher wear rate compared to feldspathic ceramic. This is reflective of the present study, which showed higher roughness in polymer-infiltrated ceramics compared to feldspathic ceramics, and stained specimens showing higher roughness than polished specimens. From a clinical perspective, an increase in roughness of restorations with continued function can result in loss of gloss and translucency [24]. In addition, a rough surface is susceptible to plaque accumulation and bacterial growth, leading to tooth decay and periodontal disease [24,30].

On the other hand, staining covers the final surface, for better appearance and gloss effect, and long-term retention with glazing [24,25]. Therefore, it can be appreciated that the present study demonstrated higher values of gloss loss and surface roughening in the stained groups compared to the polished groups. This signifies the importance of filler size that undergoes homogenous abrasion and maintains the polishing effect compared to the stain.

In addition to toothbrush abrasion and dietary acids, restorations are exposed to dynamic continuous chewing forces from opposing teeth and restorations (metals, ceramic, and resin) in the oral environment. The changes in surface properties (gloss and roughness) of resin-infiltrated ceramics in the presence of such conditions are unknown. Further studies are recommended to investigate the long-term effects of occlusal chewing forces against different surfaces and dietary agents on surface roughness, color stability, gloss, and topography of resin-infiltrated ceramics. Nevertheless, within the limitations of this study, a significant increase in the surface roughness irrespective of different storage mediums was observed among polymer-infiltrated ceramics compared to feldspathic glass ceramic; however, loss of gloss was higher among polymer-infiltrated ceramics compared to conventional glass ceramics.

## 5. Conclusions

Abrasion increased surface roughness and compromised surface gloss among all polymer-infiltrated ceramic materials. Surface staining presented higher surface roughness compared to the polished surfaces, irrespective of the storage medium (citric acid or water). Surface roughness among polymer-infiltrated ceramics was significantly higher with toothbrush abrasion as compared to conventional glass ceramic. Most of the polymer-infiltrated ceramics (except SH) showed higher loss of gloss from abrasion compared to conventional glass ceramic. Overall, conventional glass ceramic showed significantly better resistance to roughness and loss of gloss due to toothbrushing abrasion as compared to polymer-infiltrated ceramics.

## Figures and Tables

**Table 1:** Material composition and manufacture details.

Material	Symbol	Shade/Block	Manufacturer	Composition
Vitablocs Mark II	VM2	(2M2/l14)	VITA Zahnfabrik (Bad Sackingen, Germany)	>20 wt.% feldspathic particles (average size of the particle 4 μm). 80 wt.% of the glass-matrix
Lava Ultimate Restorative	LU	(A2-HT/14L)	3M ESPE (MN, USA)	80 wt.% (65 vol%) of nanoceramic particles containing zirconia filler (4-11 nm), silica filler (20 nm) and aggregated zirconia/silica cluster filler) 20 wt.% (35 vol%) of highly cross-linked (methacrylate-based) polymer matrix
Vita Enamic	EN	(2M2-HT/Em-14)	Vita Zahnfabrik (Bad Sackingen, Germany)	86 wt.% of feldspathic ceramic base 14 wt.% of acrylate polymer networks (infiltrated into feldspathic ceramic base)
Shofu HC	SH	(A2-HT/14L)	Shofu Inc (Kyoto, Kyoto, Japan)	61 wt.% of silica-powder, zirconium silicate and micro fumed silica mixture 39 wt.% mixture of UDMA and TEGDMA
Crystal Ultra	CU	C-Block 15,5 x38.8 A2	Digital Dental (Scottsdale, AZ, USA)	70 wt.% of ceramic-like inorganic silicate glass filler particles (average particle size 0.8 mm (range 0.2-10.0 mm)) 30 wt.% of highly cross-linked polymer blends (Bis-GMA, UDMA, and BUDMA)

**Table 2:** Staining procedure for study materials.

Group	Instruments and Technique
SH, LU and CU groups	Each surface was coated with an adhesive (20 s) (Scotchbond Universal Adhesive; 3M ESPE, MN, USA) followed by air drying (5 s) and light polymerization (20 s) (Elipar Freelight 2, 3M ESPE, MN, USA)
EN group	Each block was etched with hydrofluoric acid (5%) for 60 s and rinsed for 15 s. Preceding the etching, the surface residue was cleansed with a surface cleaner (VitaVM LC Cleaner; VITA Zahnfabrik, Bad Sackingen, Germany) and silanized (Ceramic Primer II; GC Corp, Tokyo, Japan). The glazing agent (Vita Enamic Glaze; VITA Zahnfabrik, Bad Sackingen, Germany) was lastly applied and polymerized as previously mentioned
VM2 group	The glaze powder (Vita Akzent Plus Glaze Powder, VITA Zahnfabrik, Bad Sackingen, Germany) is mixed with the liquid (Vita Akzent Plus Glaze Fluid, (VITA Zahnfabrik Bad Sackingen, Germany) and applied using a microbrush. Consequently, the surface glaze was fired at 4-min heating cycle with 80°C/min temp increase rate; 950°C firing temp for 1 min.

**Table 3:** Mean, standard error among study groups for surface gloss loss (ΔE*_SCE-SCI_).

Study Groups	Stained-Water ΔE	Stained-Acid ΔE	Polished-Water ΔE	Polished-Acid ΔE	*p* Value ^$^	Post Hoc Comparison ^§^
VM	3.03 (0.35)	2.99 (0.42) *	2.2 (0.51) *	2.2 (0.68) *	<0.01	A
LU	3.8 (0.55)	3.5 (0.59)	3.7 (0.85)	3.4 (0.21)	0.616	B
EN	4.27 (0.84)	4.3 (1.00) *	3.5 (0.18)	3.5 (0.35)	<0.01	B
SH	2.6 (0.26) *	2.04 (0.42)	2.7 (0.30) *	2.6 (0.41)	0.053	A
CU	4.3 (0.47)	4.2 (0.76) *	3.4 (0.33) *	3.77 (0.17) *	<0.01	B

* denote significant effect of toothbrush abrasion by comparing the gloss of specimens before and after abrasion (*t*-test). ^$^ showing overall effect of storage and surface treatment using ANOVA (*p* value). ^§^ represents results of multiple comparisons test among the material groups for surface gloss loss; dissimilar capital alphabets represent statistically significant difference.

**Table 4:** Comparison between individual subgroups among the materials for surface gloss (ΔE*_SCE-SCI_).

Study Groups	VM (ΔE)	LU (ΔE)	EN (ΔE)	SH (ΔE)	CU (ΔE)	*p* Value ^$^	Post Hoc Comparison ^§^
Stained—Water	3.03 (0.35)	3.8 (0.55)	4.27 (0.84)	2.61 (0.26) *	4.31 (0.47)	<0.001	A
Stained—Acid	2.99 (0.42) *	3.5 (0.59)	4.30 (1.00) *	2.04 (0.42)	4.23 (0.76) *	<0.001	A
Polished—Water	2.20 (0.51) *	3.7 (0.85)	3.50 (0.18)	2.70 (0.30) *	3.40 (0.33) *	<0.001	A
Polished—Acid	2.23 (0.68) *	3.4 (0.21)	3.53 (0.35)	2.64 (0.41)	3.77 (0.17) *	<0.01	A
Overall Mean	2.61 (0.49) *	3.65 (0.55)	3.91 (0.59)	2.49 (0.34)	3.92 (0.43) *	<0.01	/

* denote significant effect of toothbrush abrasion by comparing the gloss of specimens before and after abrasion (*t*-test). ^$^ showing effect of material type using ANOVA. ^§^ represents results of multiple comparison test among the surface treatment/storage groups for surface gloss loss; dissimilar capital alphabets represent statistically significant difference.

**Table 5:** Mean and standard error among study groups for surface roughness (Ra, μm).

Mean	Stained-Water (Ra, μm)	Stained-Acid (Ra, μm)	Polished-Water (Ra, μm)	Polished-Acid (Ra, μm)	*p* Value ^$^	Post Hoc Comparison ^§^
VM	0.16 (0.04)	0.16 (0.047)	0.13 (0.039) *	0.134 (0.038) *	0.78	A
LU	0.29 (0.076) *	0.33 (0.080) *	0.197 (0.04) *	0.19 (0.48) *	0.01	B
EN	0.29 (0.04) *	0.28 (0.06) *	0.16 (0.05) *	0.19 (0.04) *	0.01	B
SH	0.36 (0.08) *	0.40 (0.99) *	0.21 (0.044) *	0.21 (0.022) *	0.01	B
CU	0.31 (0.05) *	0.37 (0.088) *	0.20 (0.05) *	0.21 (0.04) *	0.01	B

* denote significant effect of toothbrush abrasion by comparing the roughness of specimens before and after abrasion (*t*-test). ^$^ showing effect of storage and surface treatment using ANOVA (*p*-value). ^§^ represents results of multiple comparisons test among the surface material groups for surface roughness; dissimilar capital alphabets represent statistically significant difference.

**Table 6:** Comparison between individual subgroups among the materials for surface roughness (Ra, μm) (mean and standard error).

Mean	VM (Ra, μm)	LU (Ra, μm)	EN (Ra, μm)	SH (Ra, μm)	CU (Ra, μm)	*p* Value ^$^	Post Hoc Comparison ^§^
Stained—Water	0.16 (0.04)	0.29 (0.076) *	0.29 (0.04) *	0.36 (0.08) *	0.31 (0.05) *	0.01	A
Stained—Acid	0.16 (0.047)	0.33 (0.080) *	0.28 (0.06) *	0.40 (0.99) *	0.37 (0.088) *	0.01	A
Polished—Water	0.13 (0.039) *	0.197 (0.04) *	0.16 (0.05) *	0.21 (0.044) *	0.20 (0.05) *	0.025	B
Polished—Acid	0.134 (0.038) *	0.19 (0.48) *	0.19 (0.04) *	0.21 (0.022) *	0.21 (0.04) *	0.016	B
Overall Mean	0.145 (0.041)	0.25 (0.061)	0.23 (0.048)	0.29 (0.061)	0.27 (0.057)	<0.01	/

* denote significant effect of toothbrush abrasion by comparing the roughness of specimens before and after abrasion (*t*-test). ^$^ showing effect of material type using ANOVA (*p*-value). ^§^ represents results of multiple comparisons test among the surface treatment/storage groups for surface roughness; dissimilar capital alphabets represent statistically significant difference.

## Data Availability

The data that support the findings of this study are available from the corresponding author upon reasonable request.

## References

[1] Gracis S., Thompson V.P., Ferencz J.L., Silva N.R., Bonfante E.A. (2016). A New Classification System for All-Ceramic and Ceramic-like Restorative Materials. Int. J. Prosthodont..

[2] Lawson N.C., Burgess J.O. (2016). Gloss and Stain Resistance of Ceramic-Polymer CAD/CAM Restorative Blocks. J. Esthet. Restor. Dent..

[3] Mörmann W.H., Stawarczyk B., Ender A., Sener B., Attin T. (2013). Wear characteristics of current aesthetic dental restorative CAD/CAM materials: Two-body wear, gloss retention, roughness and martens hardness. J. Mech. Behav. Biomed. Mater..

[4] Tribst J.P., Dal Piva A.M., Werner A., Anami L.C., Bottino M.A., Kleverlaan C.J. (2020). Durability of staining and glazing on a hybrid ceramics after the three-body wear. J. Mech. Behav. Biomed. Mater..

[5] El-Damanhoury H.M., Elsahn N.A., Sheela S., Gaintantzopoulou M.D. (2021). Adhesive luting to hybrid ceramic and resin composite CAD/CAM Blocks:Er:YAG Laser versus chemical etching and micro-abrasion pretreatment. J. Prosthodont. Res..

[6] Say E.C., Yurdagüven H., Yaman B.C., Özer F. (2014). Surface roughness and morphology of resin composites polished with two-step polishing systems. Dent. Mater. J..

[7] Awad D., Stawarczyk B., Liebermann A., Ilie N. (2015). Translucency of esthetic dental restorative CAD/CAM materials and composite resins with respect to thickness and surface roughness. J. Prosthet. Dent..

[8] Heintze S., Forjanic M., Rousson V. (2006). Surface roughness and gloss of dental materials as a function of force and polishing time in vitro. Dent. Mater..

[9] Koizumi H., Saiki O., Nogawa H., Hiraba H., Okazaki T., Matsumura H. (2015). Surface roughness and gloss of current CAD/CAM resin composites before and after toothbrush abrasion. Dent. Mater. J..

[10] Kamonkhantikul K., Arksornnukit M., Lauvahutanon S., Takahashi H. (2016). Toothbrushing alters the surface roughness and gloss of composite resin CAD/CAM blocks. Dent. Mater. J..

[11] Lefever D., Perakis N., Roig M., Krejci I., Ardu S. (2012). The effect of toothbrushing on surface gloss of resin composites. Am. J. Dent..

[12] Maawadh A.M., Almohareb T., Al-Hamdan R.S., Al Deeb M., Naseem M., Alhenaki A.M., Vohra F., Abduljabbar T. (2020). Repair strength and surface topography of lithium disilicate and polymer-infiltrated ceramics with LLLT and photodynamic therapy in comparison to hydrofluoric acid. J. Appl. Biomater. Funct. Mater..

[13] Şen N., Tuncelli B., Göller G. (2018). Surface deterioration of monolithic CAD/CAM restorative materials after artificial abrasive toothbrushing. J. Adv. Prosthodont..

[14] O’Neill C., Kreplak L., Rueggeberg F.A., Labrie D., Shimokawa C.A.K., Price R.B. (2018). Effect of toothbrushing on gloss retention and surface roughness of five bulk-fill resin composites. J. Esthet. Restor. Dent..

[15] Ereifej N.S., Oweis Y., Eliades G. (2012). The Effect of Polishing Technique on 3-D Surface Roughness and Gloss of Dental Restorative Resin Composites. Oper. Dent..

[16] Da Silva E.M., Doria J., da Silva J.d.J.R., Santos G.V., Guimaraes J.G.A., Poskus L.T. (2013). Longitudinal evaluation of simulated toothbrushing on the roughness and optical stability of microfilled, microhybrid and nanofilled resin-based composites. J. Dent..

[17] Lefever D., Krejci I., Ardu S. (2014). Laboratory evaluation of the effect of toothbrushing on surface gloss of resin composites. Am. J. Dent..

[18] Eisenburger M., Addy M., Hughes J., Shellis R. (2001). Effect of Time on the Remineralisation of Enamel by Synthetic Saliva after Citric Acid Erosion. Caries Res..

[19] Alrahlah A., Khan R., Al-Odayni A.-B., Saeed W.S., Bautista L.S., Vohra F. (2020). Evaluation of Synergic Potential of rGO/SiO_2_ as Hybrid Filler for BisGMA/TEGDMA Dental Composites. Polymers.

[20] Erdemir U., Sancakli H.S., Yildiz E. (2012). The effect of one-step and multi-step polishing systems on the surface roughness and microhardness of novel resin composites. Eur. J. Dent..

[21] Pala K., Tekçe N., Tuncer S., Serim M.E., Demirci M. (2016). Evaluation of the surface hardness, roughness, gloss and color of composites after different finishing/polishing treatments and thermocycling using a multitechnique approach. Dent. Mater. J..

[22] Sharma A., Babbar A., Jain V., Gupta D. (2018). Enhancement of surface roughness for brittle material during rotary ultrasonic machining. Proceedings of the MATEC Web of Conferences.

[23] Gönülol N., Yılmaz F. (2012). The effects of finishing and polishing techniques on surface roughness and color stability of nanocomposites. J. Dent..

[24] Özarslan M.M., Büyükkaplan U.Ş., Barutcigil Ç., Arslan M., Türker N., Barutcigil K. (2016). Effects of different surface finishing procedures on the change in surface roughness and color of a polymer infiltrated ceramic network material. J. Adv. Prosthodont..

[25] Trauth K.G.S., Godoi A.P., Colucci V., Corona S.A.M., Catirse A.B.C.E.B. (2012). The influence of mouthrinses and simulated toothbrushing on the surface roughness of a nanofilled composite resin. Braz. Oral Res..

[26] Can Say E., Yurdagüven H., Malkondu Ö., Ünlü N., Soyman M., Kazazoğlu E. (2014). The effect of prophylactic polishing pastes on surface roughness of indirect restorative materials. Sci. World J..

[27] Takahashi R., Jin J., Nikaido T., Tagami J., Hickel R., Kunzelmann K.-H. (2013). Surface characterization of current composites after toothbrush abrasion. Dent. Mater. J..

[28] Lebon N., Tapie L., Vennat E., Mawussi B. (2015). Influence of CAD/CAM tool and material on tool wear and roughness of dental prostheses after milling. J. Prosthet. Dent..

[29] Dal Piva A.M.D.O., Bottino M.A., Anami L.C., Werner A., Kleverlaan C.J., Lo Giudice R., Famà F., Silva-Concilio L.R.D., Tribst J.P.M. (2021). ToothbrushingWear Resistance of Stained CAD/CAM Ceramics. Coatings.

[30] Bollenl C.M., Lambrechts P., Quirynen M. (1997). Comparison of surface roughness of oral hard materials to the threshold surface roughness for bacterial plaque retention: A review of the literature. Dent. Mater..

